# *Trichinella spiralis*-Secreted Products Promote Collagen Capsule Formation through TGF-β1/Smad3 Pathway

**DOI:** 10.3390/ijms241915003

**Published:** 2023-10-09

**Authors:** Ge Cheng, Zifang Zhang, Yixuan Wang, Youjiao Zao, Ruoqi Wang, Mengying Gao, Miaomiao Feng, Xi Zhang, Peng Jiang

**Affiliations:** Department of Pathogen Biology, School of Basic Medical Sciences, Zhengzhou University, Zhengzhou 450001, China

**Keywords:** *Trichinella spiralis*, newborn larvae, excretory–secretory (ES) products, TGF-β1/Smad3 pathway, collagen capsule formation

## Abstract

*Trichinella spiralis* (*T. spiralis*) muscle larvae colonize in the host’s skeletal muscle cells, which are surrounded by collagen capsules. The mechanism underlying muscle stage larva-induced collagen capsule formation remains unknown. To clarify the mechanism, a *T. spiralis* muscular-infected mouse model was established by a single lateral tail vein injection with 20,000 *T. spiralis* newborn larvae (NBL). The infected mice were treated with or without SB525334 (TGF-β1 receptor type I inhibitor). Diaphragms were obtained post-infection, and the expression levels of the TGF-β1/Smad3 pathway-related genes and collagen genes (type IV and VI) were observed during the process of collagen capsule formation. The changes in myoblasts under stimulation of the excretory–secretory (ES) products of NBL with or without SB525334 were further investigated. Results showed that the expression levels of type IV collagen gene, type VI collagen gene, *Tgfb1,* and *Smad3* were significantly increased in infected mice muscle cells. The expression levels of all the above genes were enhanced by the products of NBL in myoblast cells. These changes were reversed by co-treatment with SB525334 in vivo and in vitro. In conclusion, the TGF-β1/Smad3 pathway can be activated by *T. spiralis* infection in muscle cells. The activated TGF-β1/Smad3 pathway can stimulate the secretion of collagens by myocytes and plays a promoting role in the process of collagen capsule formation. The research has the limitation that the protein identification of the products of NBL has yet to be performed. Therefore, the specific components in the *T. spiralis* ES products that induce collagen synthesis should be further investigated.

## 1. Introduction

*Trichinella spiralis* (*T. spiralis*) is a meat-borne parasitic nematode. Infection occurs when the host eats meat containing infective larvae of *T. spiralis* [1,2]. Adults can release newborn larvae (NBL) that enter into circulation. NBL invade striated skeletal muscle cells and form the nurse cell–parasite complexs. The complex is surrounded by a thin capsule composed of type IV and type VI collagen [3].

The study has shown that collagen synthesis genes in infected muscles of *T. spiralis* were over-expressed. Moreover, the TGF-β1/Smad pathway-related genes (*Tgfb1*, *Smad2,* and *Smad3*) in muscles were also significantly increased [4]. Further research has found that capsule formation was initiated by excretory–secretory (ES) products of *T. spiralis* muscle larvae. ES products could also induce the high expression of *Tgfb1*, *Smad2,* and *Smad3* in fibroblast cells [4]. Transforming growth factor TGF-β1 is a multi-functional cytokine that promotes fibrosis and the synthesis of extracellular matrix (ECM) components, including collagen [5]. It can activate Smad2 and Smad3 by TGF-β receptor I (TβRI) to promote the transcription of target genes. TGF-β1/Smad3 is the classical signaling pathway that leads to fibrosis [6]. Therefore, we hypothesized that TGF-β1/Smad3 might play a role in regulating the formation of collagen capsules in *T. spiralis* infection.

To clarify the mechanism of *T. spiralis* capsule formation, the selection of the evaluation animal model is critical. The natural oral route is commonly used for the establishment of the *T. spiralis* infection model in vivo. However, the model has both intestinal and muscular phases. Adult females release NBL on the fourth day after the establishment of intestinal infection. The process can last for several days. Larvae enter skeletal muscle in batches, resulting in the asynchronous formation of collagen capsules in the muscle cells [7]. Intravenous injection of NBL can also establish an infection model successfully. Intravenous injection of NBL is performed by a single injection. Infection by this route not only eliminates the intestinal immune response but also results in the synchronous development of nurse cells [8].

*T. spiralis* may activate the TGF-β1/Smad3 pathway, increasing collagen synthesis. In this study, a *T. spiralis* muscular infected mouse model was established by a single lateral tail vein injection with 20,000 NBL. The expression levels of several genes related to collagen capsule formation and the TGF-β1/Smad3 signaling pathway-related factors (*Tgfb1* and *Smad3*) in infected skeletal muscles were measured. The gene expression levels mentioned above in myoblast cells treated with the ES products of NBL were examined.

## 2. Results

### 2.1. Synchronous Development of Collagen Capsules in Intravenously Infected Mice Muscles by T. spiralis

**Figure 1:**
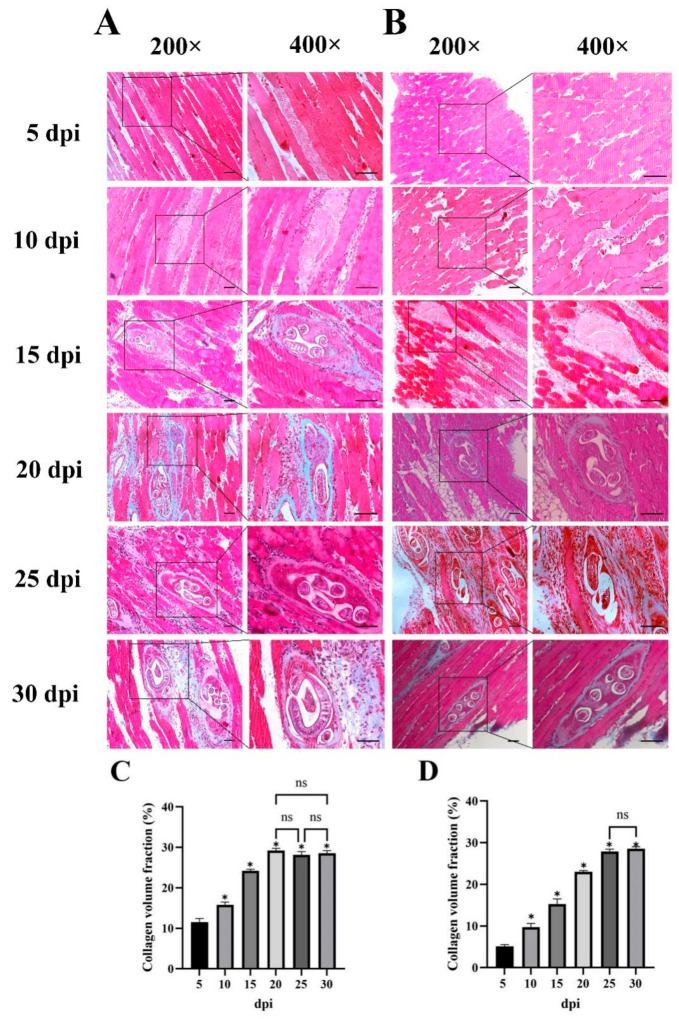
Collagen capsule development around the nurse cells in *T. spiralis*-infected diaphragms. The figure shows Masson staining images of diaphragms collected from days 5 to 30 post-infection. (**A**) the collagen capsule formation of infected mice diaphragms in intravenous infection group; (**B**) the collagen capsule formation of infected mice diaphragms in oral infection group; (**C**) the collagen volume fraction of collagen capsule in intravenous infection group; (**D**) the collagen volume fraction of collagen capsule in oral infection group. dpi, days post-infection. Scale bar: 50 µm. Results represent mean ± SD (*n* = 3). The Collagen volume fraction at 5 dpi as control group, * *p* < 0.05. ns, no statistical significance.

To better investigate the formation of the *T. spiralis* capsule, it is necessary to exclude the influence of intestinal immunity and continuous delivery of NBL to muscle for several days; the mouse-infected model by the natural oral route or intravenous injection of NBL was constructed. The diaphragm, tongue, masseter, and gastrocnemius were collected from days 0 to 30 post-infection, and the larvae were examined under a microscope. The results revealed that the diaphragm was the preferred site of colonization both by oral infection and injection of NBL (Figure S1). Then, the diaphragms were evaluated with Masson’s trichrome staining to determine the formation of a collagen capsule surrounding the nurse cell. In the intravenous infection group, although the immature larvae were observed in the diaphragm as early as 5 dpi, they kept an elongated orientation and did not invade the muscle fibers. At 10 dpi, the larvae invaded the muscle fibers, started to assume a coiled state, and began to grow rapidly. A small amount of collagen could be observed surrounding the nurse cell. At 15 dpi, collagen capsules surrounding the nurse cells were clearly distinguished in the skeletal muscle. From 20 dpi, collagen capsules became thicker, and the shape was uniform (Figure 1A). In the oral infection group, no larva was observed at 5 dpi; they appeared at 10 dpi. The larvae invaded the muscle and began to differentiate at 15 dpi, and the initial surrounding capsules were observed at 20 dpi. From 25 dpi, the collagen capsules surrounding nurse cells were clearly distinguished in the skeletal muscle, but the size was inconsistent (Figure 1B). During the infection, inflammatory cells around the collagen capsules were significantly increased at 25 and 30 dpi in orally infected mice. This phenomenon occurred in intravenous infections at 20 and 30 dpi.

### 2.2. Type IV Collagen and Type VI Collagen Were Highly Expressed in T. spiralis-Infected Muscle Tissues and Regulated by TGF-β1/Smad3 Pathway

Intravenously infected mouse models can exclude the influence of intestinal immunity, and collagen capsules develop synchronously during *T. spiralis* infection. Therefore, the intravenously infected mouse is an ideal animal model to study the mechanism underlying collagen capsule formation. To determine whether *T. spiralis* forms collagen capsules via the TGF-β1/Smad3 pathway, we infected mice by intravenous route. The expression levels of *Tgfb1*, *Smad3*, type IV collagen gene, and type VI collagen gene in diaphragms were evaluated at protein and mRNA levels using Western blot and q-PCR at different days post-infection.

**Figure 2:**
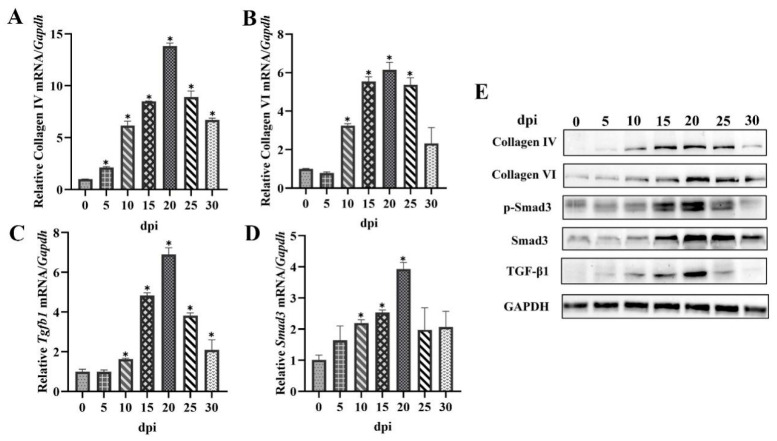
The relative expression of *Tgfb1*, *Smad3*, type IV collagen gene, and type VI collagen gene in *T. spiralis*-infected diaphragms. (**A**–**D**) the relative gene transcription levels of type IV collagen, type VI collagen, *Tgfb1,* and *Smad3* in diaphragms of *T. spiralis*-infected mice from 0 to 30 dpi. (**E**) the protein expression levels of TGF-β1, Smad3, p-Smad3, collagen IV, and collagen VI in diaphragms of *T. spiralis*-infected mice from 0 to 30 dpi; Results represent mean ± SD (*n* = 3), * *p* < 0.05.

Compared with the uninfected group, at 10 dpi, the transcription levels of type IV collagen and type VI collagen genes were significantly increased (Figure 2A,B). In addition, the gene transcription levels of type IV collagen, type VI collagen, *Tgfb1,* and *Smad3* were significantly increased as the muscle stage progressed and peaked at 20 dpi (Collagen IV: *F* = 616.197, *p* < 0.05; Collagen VI: *F* = 99.546, *p* < 0.05; *Tgfb1*: *F* = 239.672, *p* < 0.05; *Smad3*: *F* = 16.350, *p* < 0.05) (Figure 2A–D). However, the transcription levels of all genes decreased gradually at 25 dpi when compared with those at 20 dpi. To further verify the q-PCR results, we employed Western blot assay. Protein expression of type IV collagen and type VI collagen significantly increased at 10 dpi and peaked at 20 dpi (Collagen IV: *F* = 26.397, *p* < 0.05; Collagen VI: *F* = 22.764, *p* < 0.05) (Figure 2E). The protein expression of TGF-β1, Smad3, and phosphorylated Smad3 (p-Smad3) significantly increased at 10 dpi and peaked at 20 dpi (TGF-β1: *F* = 5.092, *p* < 0.05; Smad3: *F* = 3.817, *p* < 0.05; p-Smad3: *F* = 10.914, *p* < 0.05). The expression of protein reduced until nurse cells matured at 25 dpi when compared with those at 20 dpi (Figure 2E). The huge elevation of p-Smad3 indicated that the TGF-β1/Smad3 pathway was activated. These results showed that during the collagen capsule development of *T. spiralis,* the TGF-β1/Smad3 pathway was activated.

**Figure 3:**
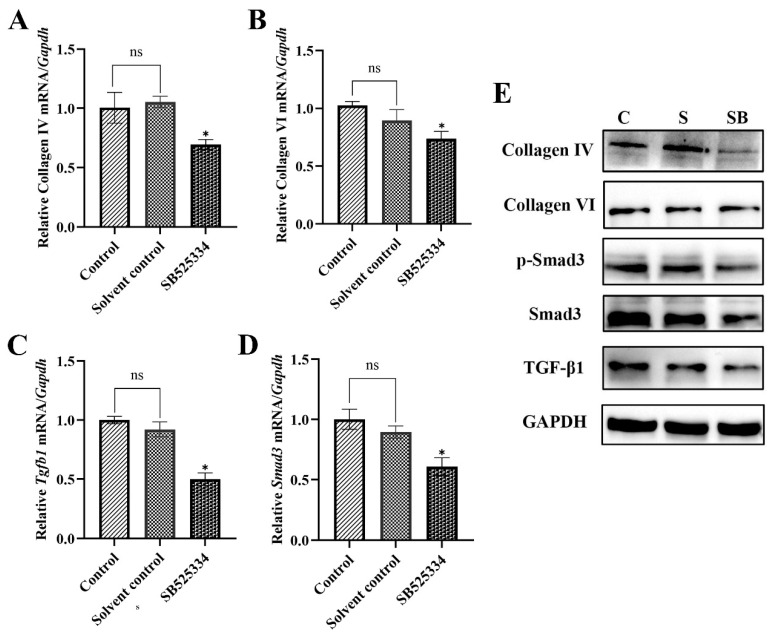
The relative expression of *Tgfb1*, *Smad3*, type IV collagen gene, and type VI collagen gene in *T. spiralis*-infected diaphragms treated with SB525334. (**A**–**D**) the relative gene transcription levels of type IV collagen and type VI collagen, *Tgfb1,* and *Smad3*; (**E**) the protein expression levels of TGF-β1, Smad3, p-Smad3, type IV collagen, and type VI collagen. C, Control group; S, Solvent group; SB, SB525334 group. ns, no statistical significance. Results represent mean ± SD (*n* = 3), * *p* < 0.05.

To further reveal the positive association between collagen capsule formation and the TGF-β1/Smad3 signaling pathway, the *T. spiralis* infected mice were treated with SB525334 to block the TGF-β1/Smad3 pathway. The diaphragms were collected at 20 dpi. The expression levels of *Tgfb1*, *Smad3*, type IV collagen gene, and type VI collagen gene in diaphragms were evaluated at protein and mRNA levels. In the inhibitor group, compared with the untreated group, the gene transcription levels of type IV collagen (*F* = 16.4, *p* < 0.05), type VI collagen (*F* = 13.343, *p* < 0.05) and *Smad3* (*F* = 24.822, *p* < 0.05) were significantly decreased. Transcription level of *Tgfb1* (*F* = 83.552, *p* < 0.05) also decreased significantly (Figure 3A–D). The Western blot also showed similar trends to those observed with q-PCR. The protein level of type IV collagen (*F* = 17.032, *p* < 0.05) was significantly lower in the inhibitor mice than in the untreated mice. The protein level of type VI collagen slightly decreased (*F* = 12.610, *p* < 0.05). In addition, the expression levels of TGF-β1 (*F* = 14.951, *p* < 0.05), p-Smad3 (*F* = 15.296, *p* < 0.05), and Smad3 (*F* = 13.219, *p* < 0.05) were significantly reduced compared with the untreated group (Figure 3E). There was no significant difference between the solvent group and the untreated group.

Our data showed a close correlation between collagen synthesis and the TGF-β1/Smad3 pathway in *T. spiralis*-infected muscle tissues, suggesting that collagen capsule formation of *T. spiralis* was promoted by the TGF-β1/Smad3 pathway.

### 2.3. Trichinella spiralis-Secreted Products Promoted Collagen Synthesis in Myoblasts through TGF-β1/Smad3 Pathway

*T. spiralis* has the ability to promote collagen capsule formation through the TGF-β1/Smad3 pathway. Studies have shown that the formation of collagen capsules is initiated by ES proteins produced by *T. spiralis* muscle larvae. To determine whether ES proteins of *T. spiralis* NBL (NBL-ESP) can enhance collagen gene expression and identify the cellular origins of capsule collagen, we evaluated the gene expression levels of *Tgfb1*, *Smad3*, type IV collagen and type VI collagen in murine C2C12 myoblasts under the stimulation of NBL-ESP at protein and mRNA levels using Western blot and q-PCR.

**Figure 4:**
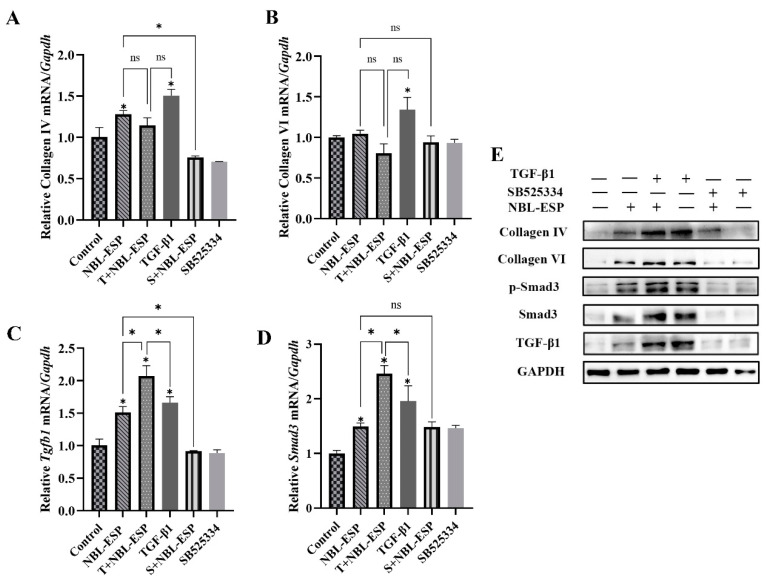
The relative expression of *Tgfb1*, *Smad3*, type IV collagen gene, and type VI collagen gene in TGF-β1 or SB525334 treated mouse myoblasts under the stimulation of *T. spiralis* NBL ES products. (**A**–**D**) the relative gene transcription levels of type IV collagen, type VI collagen, *Tgfb1,* and *Smad3*; (**E**) the protein expression levels of TGF-β1, Smad3, p-Smad3, type IV collagen, and type VI collagen. NBL-ESP, excretory-secreted products of NBL treated cells; T + NBL-ESP, TGF-β1 treated cells under the stimulation of NBL-ESP; TGF-β1, TGF-β1 treated cells; S + NBL-ESP, SB525334 treated cells under the stimulation of NBL-ESP; SB525334, SB525334 treated cells. ns, no statistical significance; Results represent mean ± SD (*n* = 3), * *p* < 0.05.

In order to determine the optimal culture concentration, C2C12 cells were treated with various concentrations of ES products. The results showed that the concentration of ES products at 5 μg/mL did not affect the proliferation of C2C12 cells (Figure S2). Finally, the cells were treated with TGF-β1 (10 ng/mL) or SB525334 (10 μM) under the stimulation of ES products (5 μg/mL). Compared with the untreated group, the gene expression levels of type IV collagen (*F* = 59.406, *p* < 0.05), *Tgfb1* (*F* = 76.779, *p* < 0.05), and *Smad3* (*F* = 37.232, *p* < 0.05) were significantly increased under the stimulation of NBL-ESP. The gene expression level of type VI collagen was increased but not significant. The gene expression levels of type VI collagen (*t* = −3.825, *p* < 0.05), type IV collagen (*t* = −5.265, *p* < 0.05), *Tgfb1* (*t* = −8.436, *p* < 0.05), and *Smad3* (*t* = −5.750, *p* < 0.05) were significantly increased under the stimulation of TGF-β1. TGF-β1 further increased the expression of *Tgfb1* (*t* = −5.032, *p* < 0.05) and *Smad3* (*t* = −10.303, *p* < 0.05) promoted by NBL-ESP. The enhanced expression levels of type IV collagen and *Tgfb1* promoted by NBL-ESP were inhibited by TGF-β1 receptor inhibitor (Figure 4A–D). Compared with untreated group, the protein expressions of type IV collagen (*F* = 4.211, *p* < 0.05), type VI collagen (*F* = 13.806, *p* < 0.05), TGF-β1 (*F* = 30.298, *p* < 0.05), Smad3 (*F* = 8.110, *p* < 0.05), and p-Smad3 (*F* = 397.67, *p* < 0.05) in the C2C12 cells increased significantly under the stimulation of NBL-ESP. Although TGF-β1 further enhanced the protein expression under the stimulation of NBL-ESP, the difference was not significant compared to NBL-ESP treatment alone. The enhanced expression promoted by NBL-ESP was inhibited by TGF-β1 receptor inhibitor (Figure 4E). The above results showed that NBL-ESP could promote collagen synthesis in myoblast cells through the TGF-β1/Smad3 pathway.

## 3. Discussion

*T. spiralis* is an intracellular nematode that completes its life cycle by encysting in the striated muscle tissues of the host. Once the larvae invade skeletal muscle cells, they reenter muscle cells into the cell cycle and transform them into nurse cells, which recruit nutrients for the growth and maintenance of the larvae. The nurse cell–muscle larva complex fully develops during 15–20 days [9]. A collagen capsule usually surrounds those transformed nurse cells, while the mechanism of collagen capsule formation is unclear. Establishing an animal model is very important to determine the mechanisms underlying collagen capsule formation. Oral infection is the natural infection route for *T. spiralis*. However, infection by this route can cause an intestinal immune response. Intestinal infection promotes myositis [7]. In addition, the delivery of NBL to muscle lasts a long time, resulting in the asynchronous development of larvae, and the conditions mentioned above increase the difficulty of collagen capsule formation research. In contrast, intravenous infection is performed with a single injection of NBL. This infection by vein not only eliminates the intestinal immune response but also makes nurse cells develop synchronously. In this study, we established *T. spiralis* infected mice by gavage or by intravenous injection of NBL. Pathologic results showed that the development of collagen capsules in orally infected mice was delayed compared with intravenously infected mice, as worms produce NBL until 6 days after infection [10]. Nurse cells developed synchronously in the muscle tissues of mice intravenously infected. The size of nurse cells was inconsistent in the muscle tissues of orally infected mice (Figure 1). In the following experiments, we used intravenously injected mice as an animal model.

Previous research has shown that the collagen capsule of *T. spiralis* mainly consists of type IV and VI collagen. In contrast, type I collagen, a significant component of bone and most musculoskeletal structures has not been detected in collagen capsules [4]. TGF-β1 was found to promote collagen production by both epithelial and mesenchymal cells. Subcutaneous injection of TGF-β1 strongly promoted collagen accumulation [11]. TGF-β1 directly activates Smad signaling, which triggers collagen gene transcription. Smad3 is the major downstream [12]. It has been confirmed that the activation of the TGF-β signaling pathway stimulates the Smad family downstream via phosphorylation [13]. TGF-β1 induced collagen I expression in fibroblasts has been highlighted; however, the studies on the role of TGF-β1 in type IV collagen and type VI collagen are not very much. Research showed that TGF-β1 was significantly increased during *T. spiralis* infection [14]. To clarify whether TGF-β1/Smad3 is involved in collagen capsule formation by *T. spiralis*, *Tgfb1*, *Smad3*, type IV collagen gene expression levels, and type VI collagen gene expression level in diaphragms at different days post-infection were examined. The results showed that the transcription levels of type IV collagen gene, type VI collagen gene, *Tgfb1,* and *Smad3* were significantly increased at 10 dpi and peaked at 20 dpi (Figure 2A–D). The transcription levels of all the above genes decreased gradually at 25 dpi compared to those at 20 dpi. A Western blot assay also showed similar trends to those observed with q-PCR. Protein expression levels of type IV collagen, type VI collagen, TGF-β1, Smad3, and p-Smad3 increased at 10 dpi and peaked at 20 dpi (Figure 2E). The nurse cell formation occurs over 20 days period from the initial larval invasion [15]. The thick capsule surrounding the nurse cell is mature at 20 dpi; therefore, the expression of collagen genes reached the peak. The expression of *Tgfb1* and *Smad3* had a similar trend. Our results showed that the collagen capsule development of *T. spiralis* is related to the TGF-β1/Smad3 pathway.

To verify the results further, we treated *T. spiralis*-infected mice with TGF-β1 receptor inhibitor SB525334 to generate TGF-β1/Smad3-blocked mice. SB525334 is a potent TβRI inhibitor that can block TGF-β-induced Smad activation and decrease collagen production [16]. The expression levels of *Tgfb1*, *Smad3*, type IV collagen, and type VI collagen in the diaphragm were evaluated. Results showed that the gene transcription levels of type IV collagen, type VI collagen, and *Smad3* significantly decreased (Figure 3A–D). Western blot also showed similar trends to those observed with q-PCR. The protein levels of type IV collagen, type VI collagen, p-Smad3, and Smad3 were inhibited by SB525334 (Figure 3E). In addition, our data showed that SB525334 suppressed the expression levels of *Tgfb1* at protein and mRNA. This finding is explained by the fact that SB525334 inhibits the TGF-β1-induced activation of Smad3, leading to suppression of autoinduction of TGF-β1 [17]. It is further believed that *T. spiralis* infection activated the TGF-β1/Smad3 pathway, which induced type IV collagen and type VI collagen production and promoted the formation of collagen capsules.

*T. spiralis* can promote collagen capsule formation through the TGF-β1/Smad3 pathway. Studies have shown that the ES proteins produced by *T. spiralis* muscle larvae initiated collagen capsule formation after being secreted into host muscle cells. The gene expression level of type I collagen was significantly increased in mouse fibroblasts treated with muscle larvae ES proteins [4]. However, previous studies indicated that the collagen capsule was mainly composed of type IV collagen and type VI collagen, and fibroblast collagens (types I and III) were not components [3,4]. In addition, muscle larvae were isolated from *T. spiralis*-infected mice at 28 dpi or more, and ES proteins from cultured muscle larvae were obtained according to the previously reported method [18]. Therefore, the ES proteins produced by *T. spiralis* muscle larvae used for capsule formation research are significantly later than the collagen capsules’ development. *T. spiralis* starts forming collagen capsules once they invade the muscle cells. NBL is the first stage of *T. spiralis* to contact with the host muscle cells. A study investigating the nurse cell formation process showed that NBL of *T. spiralis* started to secret proteins until the 7th day after infection, and the onset of host type IV collagen and type VI collagen mRNA synthesis was also between days 7 and 8 [9]. Another research has shown that ES products of NBL could promote the synthesis of type IV collagen by fibroblasts [19]. The questions of whether ES products of *T. spiralis* NBL have a role in collagen capsule formation and the cellular origins of collagen capsules are still unanswered. In our study, the expression levels of *Tgfb1*, *Smad3*, type IV collagen gene, and type VI collagen gene in NBL-ESP-treated murine C2C12 myoblasts were evaluated, and it was found that the gene expression levels of type IV collagen, *Tgfb1,* and *Smad3* were significantly increased. The corresponding protein production was significantly elevated, including type VI collagen and p-Smad3. The enhanced gene expression levels of type IV collagen, *Tgfb1,* and corresponding protein, including type VI collagen and p-Smad3 promoted by NBL-ESP, were inhibited by TGF-β1 receptor inhibitor (Figure 4). Our findings indicated that ES products of *T. spiralis* NBL resulted in the activation of the TGF-β1/Smad3 signaling pathway to promote type IV collagen synthesis in host skeletal muscle cells. The effect of ES products from NBL on the synthesis of type VI collagen was relatively small. Is this related to changes in the secretion of larvae during dynamic development or other regulatory mechanisms involved in the synthesis of collagen VI [20,21]?

Although the ES products of NBL were obtained in this study, the protein identification has yet to be performed. The composition of ES products still needs to be determined. Further studies will be needed to identify the collagen-inducing factors from ES products.

## 4. Materials and Methods

### 4.1. Parasites and ES Products

The *T. spiralis* strain (ISS534) used in this study was isolated from a naturally infected pig and passaged in mice in our laboratory. *T. spiralis* infective larvae and NBL were recovered from mice as previously described [8]. ES products of NBL were obtained from the cultures of NBL as described previously [19]. In brief, NBL were incubated in Roswell Park Memorial Institute-1640 (RPMI 1640, Gibco, Carlsbad, CA, USA) for 24 h and then removed by centrifugation at 5000× *g* for 10 min. The supernatant was passed through a sterile 0.22 μm filter. The filtrate was centrifugated at 4000× *g* and 4 °C. The protein concentration was quantified using the BCA method (Boster, Wuhan, China) and frozen at –80 °C until used.

### 4.2. Animal Experiments

Female BALB/c mice and Kunming mice (6~8-week-old) were purchased from Henan Provincial Experimental Animal Center (Zhengzhou, China) and maintained in individual ventilated cage (IVC, Suzhou, China).

The mice were classified into three groups as follows (3 mice in each experiment): negative control group, oral infection group, and intravenous infection group. For oral infection group, 300 infective larvae were administered by gavage. For intravenous infection group, mice were given a single injection of 20,000 NBL suspended in 200 μL of serum-free RPMI-1640 in tail vein. Parasite doses delivered by the two routes were calibrated to produce similar muscle burdens [7]. The mice were euthanized at 0, 5, 10, 15, 20, 25, and 30 days post-infection (dpi), and the muscle tissues were harvested for further analysis.

Subsequently, TβRI inhibitor SB525334 (Selleck, Houston, TX, USA) was applied in intravenous infection group. Considering that the solvent of SB525334 included corn oil and 0.5% DMSO, the mice were divided into 3 experimental groups: untreated group, solvent group, and inhibitor group. In the solvent and inhibitor group, after 24 h of infection with NBL, oral gavage with solvent or SB525334 (10 mg/kg/day) was performed every other day until the 20th day post-infection [16]. The mice were sacrificed at 20 days post-infection, and the muscle tissues were harvested for further analysis.

All the animal experiments in this study were carried out in accordance with the Provision and General Recommendation of Chinese Experimental Animals Administration Legislation.

### 4.3. Histological Analysis of the Diaphragm

The diaphragms were fixed in 4% paraformaldehyde, embedded in paraffin, and sectioned at 6 µm. To visualize collagen fibers, the sections were evaluated with Masson’s trichrome staining (Solarbio, Beijing, China). The collagen fibers were stained blue. Cytoplasm and muscles were stained purple-red. The Collagen volume fraction was expressed as the percentage of a capsule area that was stained with Masson’s trichrome staining [22]. The collagen area around a single capsule was measured using Image J software (1. 53q). The calculation formula for Collagen volume fraction is as follows: Collagen volume fraction = Collagen positive area/area of a single capsule × 100%. Means were calculated from 6 nurse cells in each section at a magnification of 200×.

### 4.4. Co-Culture of Myoblasts with TGF-β1 or SB525334 under Stimulating of T. spiralis ES Products

To compare the expression levels of collagen and TGF-β1 signal pathway-related genes, mouse-striated muscle myoblast C2C12 (ATCC, Manassas, VA, USA) was used and verified free of mycoplasma and bacterial contamination. 1.5 × 10^6^ cells were seeded into a 6-well plate in RPMI 1640 with 10% Fetal Bovine Serum (FBS, Gibco, Carlsbad, CA, USA) and incubated at 37 °C with 5% CO_2_ overnight. After discarding the culture medium, C2C12 cells pre-treated with TGF-β1 (Novoprotein, Suzhou, China) or SB525334 were stimulated with ES products for 12 h at 37 °C. In order to determine the optimal culture concentration, C2C12 cells were treated with various concentrations of ES products (0, 2.5, 5, 7.5, 10, and 15 μg/mL) for 12 to 48 h in 37 °C incubator with 5% CO_2_. The cell proliferation was analyzed by CCK8 assay (Glpbio, Montclair, CA, USA).

### 4.5. Quantitative Real-Time PCR (q-PCR) Analysis

The total RNA of diaphragm tissues and C2C12 myoblasts was extracted by Trizol reagent (Takara, Dalian, China), and cDNA was prepared using reverse transcriptase (Novoprotein, Suzhou, China). Relative gene expression levels of type IV Collagen, type VI Collagen, *Smad3,* and *Tgfb1* were determined by q-PCR (Vazyme, Nanjing, China). Relative expression levels of the genes were calculated as ratios to *Gapdh*. The primer sequences used are listed in Table 1 [4,23,24].

### 4.6. Western Blot Analysis

The harvested myoblasts and diaphragms were lysed in ice-cold RIPA buffer (Solarbio, Beijing, China) containing protease inhibitor (Boster, Wuhan, China). The lysates were centrifuged at 12,000× *g* for 10 min at 4 °C. The supernatants were collected for protein quantification using the BCA method. Proteins were mixed with 5× loading buffer and heated in boiling water for 5 min. The proteins were separated with 10% SDS-PAGE and transferred onto 0.45 µm PVDF membranes. The membranes were blocked with 5% skim milk for 30 min at room temperature and then incubated with primary antibodies overnight at 4 °C. The membranes were then incubated with HRP-labeled secondary antibody (zhuangzhibio, Xi’an, China) for 1 h at 37 °C. The membranes were detected using ECL and evaluated using Image J software(1.53q). Primary antibodies were as follows: anti-collagen IV (Affinity, Jiangsu, China), anti-collagen IV (Abcam, Cambridge, MA, USA), anti-TGFβ1 (Abcam), anti-Smad3 (Abcam), and anti-p-Smad3 (Abcam).

### 4.7. Statistical Analysis

Statistical analysis was performed with GraphPad Prism 9 software (9.4.0). The data were expressed as mean ± SD. One-way ANOVA test was performed for comparisons among multiple groups, and independent sample *t*-test was used for comparison between the two groups. *p* values less than 0.05 were considered statistically significant.

## 5. Conclusions

In conclusion, our data indicate that the intravenously infected mouse model has synchronously developed nurse cells and is an ideal animal model for the study of collagen capsule formation in *T. spiralis* infection. In intravenously infected mice, ES products of *T. spiralis* NBL activate the TGF-β1/Smad3 signaling pathway, and the activated TGF-β1/Smad3 pathway can stimulate the secretion of collagens by myocytes and plays a promoting role in the process of collagen capsule formation. These findings contribute to the understanding of collagen capsule formation and can also provide a new strategy for the treatment of human trichinellosis. The research has some limitations in that the protein identification has yet to be performed, and the sample sizes of the experimentally infected mice were small. That is important for our findings, and therefore, the specific components in the *T. spiralis* ES products that induce the collagen capsule synthesis should be further investigated.

## Figures and Tables

**Table 1:** Primer sequences for q-PCR.

Primer	Primer Sequences (5’-3’)
Collagen IV(*Col4a1*)-Forward	CCCGGAGTTCCAGGATTTCA
Collagen IV(*Col4a1*)-Reverse	TTAGGGCCGGGTACACCTTG
CollagenVI (*Col6a2*)-Forward	AAGGGTGTGCCTGGCTTCAAG
CollagenVI (*Col6a2*)-Reverse	CCAGTTTGCCCTTCTGTCCATC
*Tgfb1*-Forward	GTGTGGAGCAACATGTGGAACTCTA
*Tgfb1*-Reverse	TTGGTTCAGCCACTGCCGTA
*Smad3*-Forward	GAGTAGAGACGCCAGTTCTACC
*Smad3*-Reverse	GGTTTGGAGAACCTGCGTCCAT
*Gapdh*-Forward	TACCCCCAATGTGTCCGTC
*Gapdh*-Reverse	AAGAGTGGGAGTTGCTGTTGAAG

## Data Availability

Not applicable.

## Supplementary Materials

The following supporting information can be downloaded at: https://www.mdpi.com/article/10.3390/ijms241915003/s1.

## Abbreviations

TGF-β1: transforming growth factor-beta 1; *Smad3: mothers against decapentaplegic homolog 3*; NBL: *newborn larvae;* ES: excretory–secretory; ECM: extracellular matrix; RPMI: Roswell park memorial institute; DMSO: dimethyl sulfoxide; *Gapdh*: glyceraldehyde-3-phosphate dehydrogenase; RIPA: radio immunoprecipitation assay; SDS-PAGE: sodium dodecyl sulfate-polyacrylamide gel electrophoresis; PVDF: polyvinylidene fluoride; HRP: horseradish peroxidase; ECL: enhanced chemiluminescence; SD: standard deviation; ANOVA: analysis of variance; NBL-ESP: ES proteins of *T. spiralis* NBL.
