# Supplementary figures and images for: Trichinella spiralis-Secreted Products Promote Collagen Capsule Formation through TGF-β1/Smad3 Pathway
Source: Int J Mol Sci. 2023 Oct 9;24(19):15003. doi: 10.3390/ijms241915003 (PMC10573566; doi:10.3390/ijms241915003)

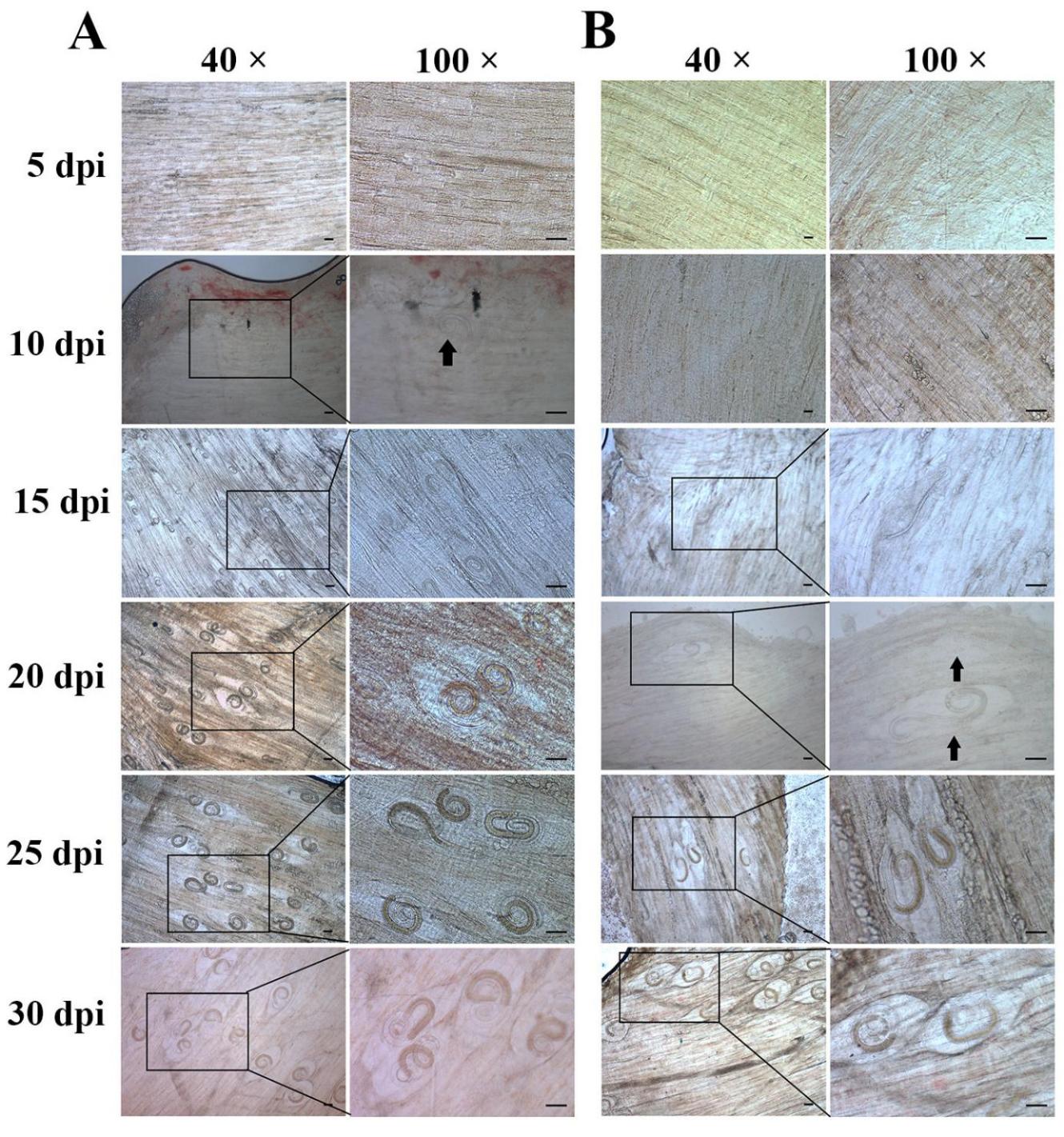

Supplement: Supplementary file 1 [file ijms-24-15003-s001.zip › Supplementary Files/Figure S1.jpg]

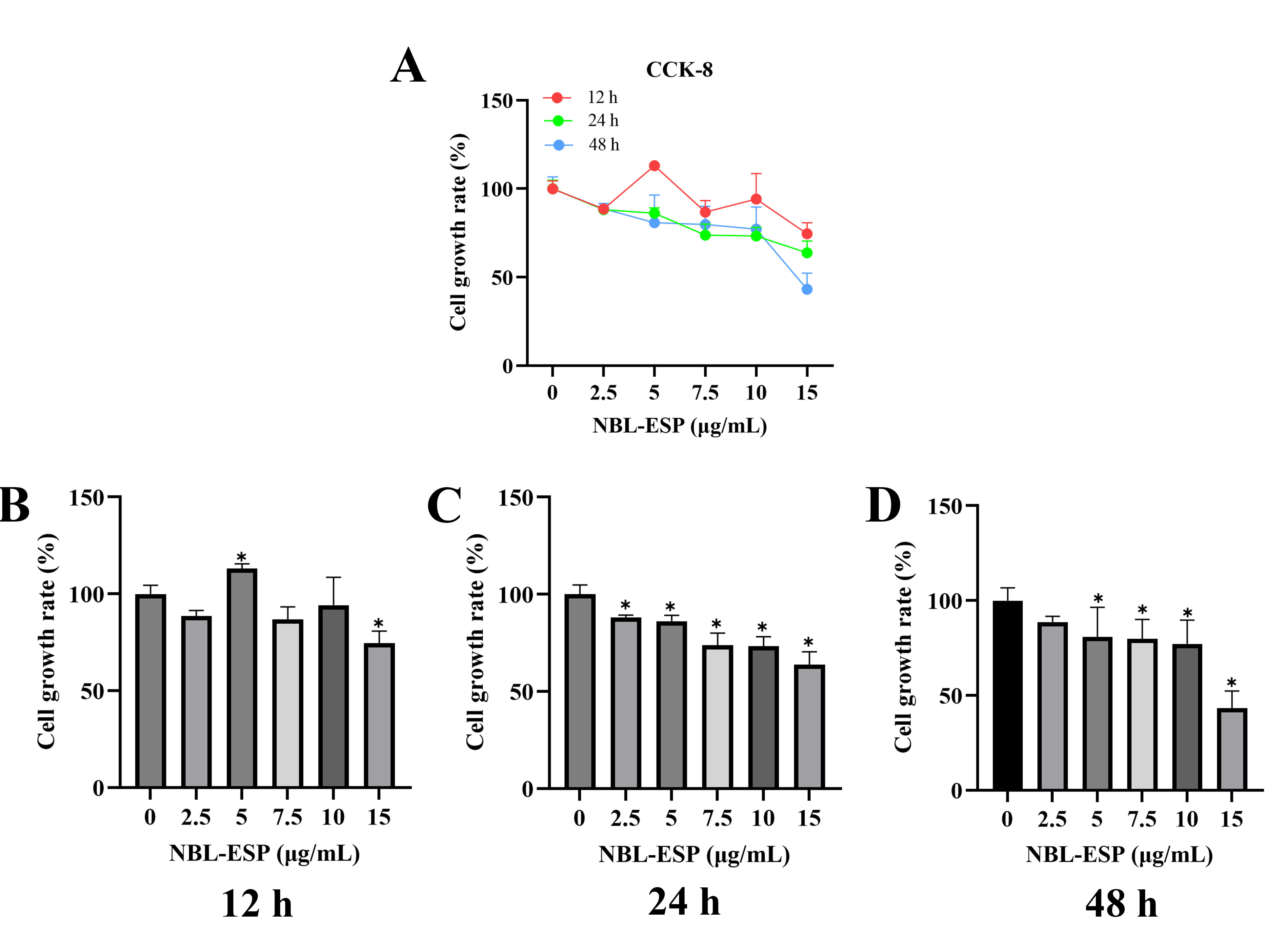

Supplement: Supplementary file 1 [file ijms-24-15003-s001.zip › Supplementary Files/Figure S2.jpg]
